# Exploration of Anesthetic Strategies for Transcatheter Tricuspid Valve Intervention

**DOI:** 10.31083/RCM44691

**Published:** 2026-01-08

**Authors:** Shuantong Lin, Yulong Guan

**Affiliations:** ^1^Department of Anesthesiology, Eastern Bund Healthcare, 202162 Shanghai, China; ^2^Department of Extracorporeal Circulation, Fuwai Hospital, Chinese Academy of Medical Sciences & Peking Union Medical College, 100037 Beijing, China

**Keywords:** tricuspid regurgitation, transcatheter tricuspid valve interventions, anesthesia

## Abstract

Tricuspid regurgitation (TR) is a critical factor in the progression of right heart failure. Although conventional open surgery remains the definitive treatment, the application of this technique is significantly limited in older and high-risk patients due to frequent comorbidities, including impaired right ventricular functional reserve, pulmonary hypertension, and multi-organ dysfunction, which lead to substantially increased surgical risks. Transcatheter tricuspid valve intervention (TTVI), which achieves anatomical correction through minimally invasive approaches, has emerged as an effective alternative strategy for patients deemed ineligible for surgery. During these procedures, anesthesiologists face three core challenges: susceptibility to acute changes in the preload of the right ventricle, a high risk of circulatory collapse (particularly in functional TR with right ventricular decompensation), and the precise integration of intraoperative transesophageal echocardiography (TEE) with hemodynamic monitoring. Consequently, anesthesiologists who become experts in the pathological staging of TR, key points of image-guided device implantation, and warning indicators of circulatory collapse can help maintain perioperative stability. Moreover, gaining a thorough understanding of the pathological progression of tricuspid valve disease, improving the assessment of right heart function, and optimizing the TTVI process and management capabilities are crucial for improving patient outcomes. Thus, establishing a perioperative anesthetic strategy focused on right heart protection may reduce cardiovascular-related complications and all-cause mortality.

## 1. Introduction

Often referred to as the ‘forgotten valve’ due to its location on the venous side of the heart, the significance of the tricuspid valve is frequently overlooked. Tricuspid regurgitation (TR) often coexists with left heart disease or pulmonary vascular pathology and is mistakenly perceived as a secondary ‘innocent bystander’, which leads to the neglect of its independent pathological importance. In the absence of left heart disease, the typical symptoms of severe TR (e.g., edema, fatigue, and reduced exercise tolerance) are often attributed to aging, resulting in delayed diagnosis and treatment. Patient survival progressively shortens as TR severity increases. With the intensification of population aging, the prevalence of TR is increasing, significantly impacting patient prognosis. Traditional treatment primarily involves surgical intervention, which is highly invasive and carries substantial risks. For patients with severe TR, the perioperative mortality rate can reach up to 10%. Given the limitations associated with the high risks of surgery, transcatheter tricuspid valve intervention (TTVI), which offers a safer profile, holds promising prospects for application. Recent breakthrough advancements in TTVI are rapidly reshaping the treatment landscape for TR. However, anesthetic management during TTVI faces three core challenges: narrowed hemodynamic tolerance due to right ventricular volume overload from TR; the high risk of acute circulatory failure triggered by maneuvers, especially in functional TR with right ventricular decompensation; and the need for precise integration of transesophageal echocardiography (TEE) with hemodynamic data for optimal decision-making. This article focuses on the pathophysiology of TR and perioperative management strategies for TTVI, aiming to provide an evidence-based intervention framework for anesthesiologists to reduce surgical risks and improve patient outcomes.

## 2. Overview of TR

As the largest cardiac valve, the tricuspid valve exhibits significant anatomical heterogeneity. According to the novel TEE classification standard established by Hahn *et al*. in 2021 [1], its leaflet morphology can be categorized into four types: Type I (classic trileaflet, 54%), Type II (bileaflet), Type III (quadrileaflet, including IIIA double anterior leaflet, IIIB double posterior leaflet, and IIIC double septal leaflet subtypes), and Type IV (multileaflet). Only half of the patients have the classic trileaflet structure (Type I), while approximately 39% present with a quadrileaflet morphology, with Type IIIB (double posterior leaflet) being the most common at 32.1% [1]. Compared to mitral valve leaflets, tricuspid leaflets are thinner, anchored to the right ventricular papillary muscles by chordae tendineae, and the annulus is a dynamic non-planar structure, with a baseline diameter of (28 ± 5 mm) and an area of (11 ± 2 cm^2^) [2]. This anatomical configuration renders the tricuspid valve susceptible to the motion of the right ventricular free wall and the state of the interventricular septum, making it highly sensitive to volume load. Tricuspid annular area dilation exceeding 40% can induce significant TR, a threshold markedly lower than the 75% required for mitral regurgitation [3]. Furthermore, a tricuspid annular diameter exceeding 34 mm in diastole or 32 mm in systole serves as a sensitive predictor of TR progression. Notably, morphological predictors of TR progression are disease-specific: in pulmonary hypertension, it primarily manifests as right ventricular enlargement/sphericity, annular dilation, and increased tenting area; whereas in atrial fibrillation, it presents as leaflet tethering, left atrial volume increase, and right ventricular remodeling. Additionally, the progression of TR after mitral valve surgery is an independent predictor of adverse clinical events [4].

## 3. Treatment of TR

The early symptoms of TR are often insidious and well tolerated. As a result, patients frequently present for surgical intervention late in the disease course, often in poor general condition and with multi-organ dysfunction, which significantly increases surgical risk. A follow-up study by Wong *et al*. [5] involving 2644 patients undergoing tricuspid valve surgery (mean follow-up of 4.9 years) reported in-hospital mortality rates of 8.7% for isolated tricuspid surgery and 8.6% for concomitant tricuspid surgery with other valve procedures, with all-cause mortality rates of 41.7% and 36.8%, respectively. Compared to tricuspid valve replacement, tricuspid valve repair significantly reduces the risk of all-cause mortality. To prevent irreversible right ventricular remodeling, early intervention for TR is necessary. For symptomatic TR patients who are unable to tolerate conventional surgery, TTVI may be considered after evaluation by a structural heart team [6]. After TTVI, tricuspid annular plane systolic excursion (TAPSE) and right ventricular fractional area change (RVFAC) significantly decreased in patients with normal right ventricle (RV) function. This decline suggests that before TTVI TAPSE and RVFAC may overestimate the real RV function, which has a negative impact on the decision for a timely tricuspid valve (TV) repair or replacement. For patients with baseline RV dysfunction (TAPSE <17 mm and RVFAC <35%), there was either no change or a slight improvement in TAPSE (from 13.2 ± 2.3 mm to 15.3 ± 4.7 mm) and RVFAC (from 29.6% ± 4.1% to 31.6% ± 8.3%). This indicates that RV dysfunction may buffer the negative effects of TR treatment on RV function. In patients with normal RV function, the decrease in TAPSE and RVFAC after TR treatment can be attributed to a phenomenon where the afterload reserve (the capacity of the RV to handle the increased afterload post-TR treatment) is overstretched, leading to functional decline. For patients with RV dysfunction, it is hypothesized that the observed increase in TAPSE may be a result of regression to the mean—essentially, a statistical tendency for extreme values (like very low baseline TAPSE) to move closer to the average post-treatment [7]. A retrospective study conducted by Tanaka *et al*. [8] compared the results of TV repair between patients with baseline RVFAC ≥35% and those with baseline RVFAC <35%, have found that by far the best results of TV repair (i.e., lowest all-cause mortality and hospitalization due to heart failure within 1 year after TV repair) were achieved in the subgroup of patients with baseline RVFAC ≥35% in which there was also a significant reduction of TAPSE and RVFAC (subgroup referred by the authors to as “RV non-responders”), whereas the worst results were observed in the subgroup of patients with baseline RVFAC <35% in which there was a significant increase in TAPSE and RVFAC (referred to as “RV responders”). Of all 204 patients included into that study, the so called “RV non-responders” in the patient group with a baseline RVFAC ≥35% were by far the largest subgroup (56% of all studied patients) and they also revealed a 73% lower incidence of composite outcome at 1 year in comparison with the so-called “RV responders” present in the same group of patients with a baseline RVFAC ≥35%. But Tanaka *et al*. [8] made the misleading error to consider the patients with reduction of TAPSE and RVFAC after TV repair as “RV non-responders” even if the reduced values of those parameters remained within the normal range, and even if the latter patient group revealed by far the best outcome at 1 year after TV repair. However, after the elimination of TR, both TAPSE and RVFAC can become more reliable parameters for estimation of RV function.

## 4. Indications for TTVI

Indications for TTVI need meet the following criteria: TR grade ≥3+; tricuspid insufficiency syndrome at least stage 2, indicating multi-system involvement; persistent clinical symptoms related to TR despite optimal medical therapy; exclusion of irreversible pulmonary arterial hypertension (PAH) with a pulmonary artery systolic pressure (PASP) of <65 mmHg (1 mmHg = 0.133 kPa); a left ventricular ejection fraction (LVEF) greater than 45%; compensated right ventricular function evidenced by a TAPSE of ≥12 mm; tricuspid anatomy that is suitable for interventional device implantation; and the patient must be classified as high-risk and unsuitable for surgical treatment by a multidisciplinary heart team. This classification must fulfill at least one of the following criteria: a Clinical Risk Score (CRS) of ≥8; a Society of Thoracic Surgeons (STS) score of ≥8; or the presence of two or more frailty indices or major organ dysfunctions that are unlikely to improve postoperatively, with a life expectancy exceeding 12 months [9, 10].

## 5. Principles for TTVI Technique Selection

Transcatheter tricuspid valve repair (TTVr) and transcatheter tricuspid valve replacement (TTVR) are the main TTVI techniques. These methods achieve anatomical correction through minimally invasive access and have become effective alternative strategies for TR patients intolerant to surgery (Table 1, Ref. [11, 12, 13, 14, 15, 16, 17]).

**Table 1.** **TTVI techniques and access route selection**.

Access route	Imaging guidance	Representative device systems	Technical principle/characteristics	Key clinical data	Advantages	Limitations and risks
Transfemoral	X-ray + TEE	TTVr: Cardioband [11] TriClip/PASCAL/DragonFly-T [12] TTVR: EVOQUE [13]	Cardioband: Annuloplasty, anchors to annulus [11] Clipping systems: Anterior-septal leaflet coaptation (“bicuspidization”) [12] EVOQUE: Self-expanding Nitinol valve, reduces paravalvular leak [13]	TRISCEND study [13] (N = 176): TR ≤ mild: 97.6% SV ↑ 10.5 ± 16.8 mL CO ↑ 0.6 ± 1.2 L/min KCCQ score ↑ 25.7 points 6MWD ↑ 56.2 m	Minimally invasive access Technically mature	Cardioband: Risk of RCA injury (5%) [11] Limited maneuvering space
Transjugular	TEE	TTVr: Trialign/K-ClipT [12] TTVR: LuX-Valve Plus [14]	Mimics Kay procedure: Plicates posterior annulus [12] LuX-Valve Plus: Unique anchor design, good TEE visibility [14]	LuX-Valve Plus study [14] (N = 14): Paravalvular leak rate: 14.29% LVEF ↑ Right ventricular reverse remodeling	Short, straight path Smaller sheath, lower vascular complication risk	Limited maneuvering space Valve size limitation (>40 mm delivery difficult) [12, 14]
Transatrial	DSA/TEE/Direct vision	TTVR: LuX-Valve [14, 16]	Septal anchor + anterior leaflet clamping Avoids reliance on radial force	TRAVEL study [16] (N = 126): TR ≤ mild: 95.2% RASV ↓ –38.3 ± 21.7 mL RVD ↓ –6.4 ± 2.3 mm 6MWD ↑ 71.3 ± 42.8 m	Suitable for large annuli (>45 mm) [15] Cases with severe calcification/failed repair	Requires thoracotomy/CPB Longer recovery More traumatic than vascular access
Transcaval Heterotopic	TEE/DSA	TTVR: TricValve (bivalve) [15] TriCento (fenestrated stent) [15]	Valve implantation in vena cava to block regurgitation Does not directly address tricuspid valve	TRICUS EURO study [17] (N = 44): 95.5% clinical improvement (KCCQ ↑ ≥15 points/6MWD ↑ ≥40 m) 63.8% hepatic vein regurgitation elimination	Simple anatomical targeting Lower procedural difficulty Suitable for very high-risk patients	Thrombosis/hepatic vein occlusion risk unknown Large individual variation in caval volume Limited long-term evidence [16]

TTVr, transcatheter tricuspid valve repair; TTVR, transcatheter tricuspid valve replacement; TEE, transesophageal echocardiography; RCA, right coronary artery; LVEF, left ventricular ejection fraction; CPB, cardiopulmonary bypass; SV, stroke volume; CO, cardiac output; KCCQ, Kansas City Cardiomyopathy Questionnaire; 6MWD, 6-minute walk distance; DSA, digital subtraction angiography; RASV, right atrial systolic volume; RVD, right ventricle diameter. ↑ means increased, ↓ means decreased.

For central TR with a coaptation gap of ≤7 mm and the absence of severe leaflet flail, transcatheter tricuspid edge-to-edge repair (T-TEER) is the preferred treatment option. In patients with severely damaged leaflets or a wide regurgitant jet, TTVR is recommended. In instances of markedly high regurgitation that preclude sufficient conventional anchoring zones, caval valve implantation may be performed [10].

## 6. Anesthetic Management

A perioperative strategy centered on right ventricular protection (RVPP) is fundamental to the hemodynamic management of patients with TR. Key components of this strategy include preoperative quantitative assessment, intraoperative multimodal monitoring, artificial intelligence (AI) early warning systems, goal-directed extubation, and postoperative anticoagulation management.

## 7. Preoperative Quantitative Assessment

Preoperative anesthetic assessment should encompass multiple indicators, including functional class as per the New York Heart Association (NYHA) classification, exercise tolerance evaluated through the 6-minute walk test, quality of life measured using the Kansas City Cardiomyopathy Questionnaire (KCCQ), volume status assessed via the edema index, and diuretic intensity determined by the diuretic index [10]. Among these, the KCCQ scale is recognized as more sensitive than the NYHA class in evaluating the severity of TR. For patients scheduled for TTVI, the TRI-SCORE system is recommended for prognostic assessment; this scoring system integrates eight clinical variables, with risk stratification categorized as follows: ≤3 points (low risk), 4–5 points (intermediate risk), and ≥6 points (high risk) [18]. Patients with TR who also have concomitant pulmonary arterial hypertension (PAH) necessitate baseline pulmonary vascular resistance (PVR) measurement via right heart catheterization (RHC) prior to TTVR [10]. A baseline PVR exceeding 4 Wood Units indicates an elevated risk of acute right ventricular afterload elevation and exacerbation of right heart failure during TTVR. TEE imaging is employed to evaluate right and left heart structures and to confirm the functional etiology of TR. A comprehensive assessment of the tricuspid annulus utilizing three-dimensional reconstructed multi-planar computed tomography (CT) scans provides critical information regarding annular measurements, right ventricular geometry, the course of the superior and inferior vena cava, and the positioning of the right coronary artery in relation to the tricuspid annulus and leaflet attachments. The combined application of TEE and cardiovascular magnetic resonance (CMR) imaging quantifies right ventricular systolic function (TAPSE ≥12 mm), diastolic function (tricuspid E/e’ ratio), and geometry (e.g., right ventricular end-diastolic volume index) [19, 20]. For patients experiencing cardiohepatic syndrome, liver stiffness measurement (LSM) via transient elastography (TE) can be utilized; an LSM greater than 8 kPa suggests a poor prognosis [21]. Furthermore, systolic ventricular interdependence and septal motion indicate that the left ventricle contributes 20%–40% of the right ventricular stroke volume [22]. Therefore, patients with TR who also suffer from concomitant left heart failure should receive active treatment for left heart failure to mitigate its impact on right ventricular output. Additionally, TR patients with atrial fibrillation should have their heart rate controlled preoperatively to 80–100 bpm. Furthermore, those indicated for a pacemaker should have one implanted prior to surgery [10].

## 8. Intraoperative Multimodal Monitoring

Standard monitoring equipment as per the American Society of Anesthesiologists (ASA) guidelines is utilized during the intraoperative period. Prior to induction, external defibrillator pads are applied, and a right radial arterial catheter is inserted for blood pressure monitoring. For patients classified as high-risk (TRI-SCORE >3), it is imperative to establish central venous access before induction. In instances where bispectral index (BIS) electrodes may interfere with regional oxygen saturation (rSO_2_) monitoring sites, priority should be given to rSO_2_ monitoring. A study conducted by Spence *et al*. [23] involving 49 patients undergoing cardiac surgery indicated that a decrease in intraoperative rSO_2_ was associated with a reduction in postoperative daily activity. Additionally, a meta-analysis by Ding *et al*. [24], which included 377 patients, demonstrated that intraoperative rSO_2_ monitoring significantly reduced the risk of postoperative neurocognitive decline and shortened hospital stays. Furthermore, research by Ju *et al*. [25] involving 394 cardiac patients revealed that a plantar rSO_2_ level below 45% increased the risk of acute kidney injury (AKI), with a longer duration correlating with a higher incidence of AKI; notably, a plantar rSO_2_ below 45% was identified as an independent risk factor for AKI. A TEE probe is routinely placed after induction, and TEE is relied upon throughout the procedure for precise guidance during TTVI. Heart rate should be maintained at 60–80 beats per minute (bpm) during valve clipping. If multiple clips are required, the heart rate should be sequentially decreased by 5–10 bpm, ensuring that it does not fall below 45 bpm intraoperatively. For valve replacement procedures, heart rate should similarly be controlled within the 60–80 bpm range [10, 22]. TEE facilitates simultaneous assessment of transvalvular pressure gradients and dynamic evaluation of right ventricular function, thereby enabling goal-directed fluid therapy (GDFT) [26, 27]. Core targets include maintaining the ratio of right ventricular end-diastolic area (RVEDA) to left ventricular end-diastolic area (LVEDA) at <0.6, stroke volume variation (SVV) at <13%, and assessing volume responsiveness based on the dynamic trend of central venous pressure (CVP), rather than its absolute value. Additionally, it is essential to ensure urine output exceeds 0.5 mL/kg/h. If the RVEDA/LVEDA ratio exceeds 0.6 and is accompanied by a decrease in cardiac output, a reverse fluid resuscitation strategy should be initiated immediately to achieve a negative fluid balance of 500–1000 mL. A propensity score-matched study conducted by Schneider *et al*. [28], which included 3153 cardiac surgery patients (1026 pairs), demonstrated that the implementation of GDFT effectively reduced excessive hemodilution in elective cardiac surgery, improved coagulation, minimized postoperative bleeding, decreased transfusion requirements, and maintained end-organ perfusion. Furthermore, a meta-analysis by Giglio *et al*. [29], involving 6325 patients, confirmed that GDFT significantly reduced postoperative complications. Throughout the procedure, baseline activated clotting time (ACT) must be strictly monitored, heparin dosage calculated precisely, and intraoperative ACT maintained between 250–300 seconds [10].

The application of 4-Dimensional Intracardiac Echocardiography (4D-ICE) in valvular interventions is becoming increasingly prevalent. This technology enables the simultaneous visualization of the heart’s three-dimensional structure along with the time dimension, allowing operators to observe valve motion and changes in blood flow visually. This capability aids in the precise anchoring of valves during procedures [30, 31]. However, despite its advantages, 4D-ICE is predominantly utilized by operators, while anesthesiologists continue to favor TEE for procedural monitoring and hemodynamic management.

## 9. AI Early Warning Systems

AI-assisted TEE decision-making primarily aims to enhance TEE image quality, identify cardiac imaging planes, and quantify and analyze cardiac function [32]. The application of AI is progressively extending to the entire cardiac care continuum, encompassing preoperative risk assessment, intraoperative planning, postoperative patient management, and outpatient remote monitoring [33]. MacKay *et al*. [34] employed AI-assisted TEE decision-making in a study involving 7106 cardiac surgery patients, demonstrating that the AI system achieved an accuracy exceeding 97% (99.4% preoperative, 97.9% postoperative) in assessing LVEF, right ventricular systolic function, and TR, with an error rate below 3% (0.6% preoperative, 2.1% postoperative). Imaging-based AI systems can elucidate the progression of cardiac function and valvular pathology [35]. Bai *et al*. [36] conducted an automated machine learning analysis of comprehensive structural and functional phenome spectra of the heart and aorta in 26,893 patients, uncovering patterns of disease phenotypes that vary by sex, age, and major cardiovascular risk factors. Seraphim *et al*. [37] utilized AI technology to measure pulmonary transit time and the pulmonary blood volume index in 985 patients undergoing myocardial perfusion assessment. This AI system automatically calculates pulmonary transit time and its derivative—the pulmonary blood volume index—from cardiac MRI, which independently predicts adverse cardiovascular outcomes. Mahayni *et al*. [38] implemented an AI early warning system based on ECG monitoring in 20,627 cardiac surgery patients, accurately identifying abnormal ECGs, predicting severe ventricular dysfunction, and assessing long-term mortality in patients with LVEF >35% undergoing valve and/or coronary artery bypass surgery. A multicenter study conducted by Vaid *et al*. [39] involving 148,227 patients demonstrated that AI could effectively predict right ventricular size and systolic function (AUC = 0.84, 95% CI 0.84–0.84). As AI systems mature in hemodynamic monitoring applications [40], they provide a novel circulatory management model for TTVI procedures.

## 10. Goal-Directed Extubation

A study by Godet *et al*. [41] on extubation following general anesthesia in 756 patients demonstrated that operating room extubation, while increasing operating room occupancy time by 8 minutes, significantly reduced respiratory-related complications, decreased the need for oxygen therapy, lowered the incidence of hypotension, and shortened the duration of stay in the post-anesthesia care unit (PACU). Additionally, a study by Teman *et al*. [42] involving 669,099 cardiac surgery patients revealed that operating room extubation was safer and more effective than fast-track extubation, leading to improved outcomes for patients undergoing coronary artery bypass grafting (CABG), aortic valve replacement (AVR), and mitral valve replacement (MVR). Given the specific nature of TTVI, operating room extubation necessitates the simultaneous fulfillment of the following criteria: recovery of right ventricular function (TAPSE ≥14 mm), controlled volume status (CVP <12 mmHg), metabolic stability (lactate ≤2 mmol/L), and independence from inhaled nitric oxide (iNO ≤5 ppm). Once heart rate increases abruptly by more than 15 beats per minute, NT-proBNP rises by more than 30%, or new ventricular arrhythmias manifest during emergence, this may indicate potential right heart failure, necessitating a delay in extubation and transfer to the intensive care unit (ICU) for further management.

## 11. Postoperative Anticoagulation Management

Postoperative management necessitates a careful balance between the bleeding risk associated with anticoagulants and the thrombotic risk posed by the valve. For patients undergoing TTVR, the preoperative anticoagulation regimen remains applicable, and oral anticoagulants (OACs) should be resumed the day following surgery. In cases where vitamin K antagonists (VKAs) are utilized, the international normalized ratio (INR) must be monitored and maintained within the range of 2.0 to 3.0. Should OACs induce valve dysfunction, their use should be discontinued, and VKA therapy should be reinstated. For patients lacking specific indications, concomitant antiplatelet therapy is advised against [43]. A study conducted by Hoerbrand *et al*. [44] involving 78 patients who underwent T-TEER revealed that, despite patients with severe TR exhibiting elevated thromboembolic and bleeding risk scores, the use of OACs significantly diminished the occurrence of major bleeding events compared to VKAs (2% vs. 21%). Moreover, no significant differences were observed in the anticoagulant effects among various OACs, and they did not elevate the risk of adverse cardiocerebrovascular events, such as stroke or heart failure. Additionally, a study by Stolz *et al*. [45] involving 141 TTVI patients indicated that both TTVR and T-TEER were linked to a postoperative decrease in platelet count. However, this transient drop in platelet levels did not result in adverse outcomes and returned to baseline levels by the time of discharge. Consequently, a vigilant approach is recommended for managing transient postoperative thrombocytopenia following TTVI. TR frequently coexists with left-sided valvular diseases, such as mitral regurgitation and aortic stenosis, as well as conditions like atrial fibrillation and heart failure. A cardiac MRI study conducted by Aabel *et al*. [46] involving 84 patients indicated that individuals with tricuspid annulus disjunction exhibited a higher incidence of mitral valve prolapse and ventricular arrhythmias. Before TTVI, it is essential to determine adjustments to medications for comorbidities, the sequence of valve interventions, and management strategies for arrhythmias through multidisciplinary consultation. The effectiveness of diuretics often obscures the severity of the disease in patients with TR; therefore, the anesthetic assessment process necessitates a comprehensive evaluation that integrates echocardiography, imaging studies, cardiac biomarkers, and laboratory tests. Patients undergoing TTVI frequently present with concomitant right heart dysfunction, PAH, and hepatic or renal impairment, resulting in significantly compromised hemodynamic reserve. During anesthetic induction, certain drugs may further depress myocardial contractility and reduce preload, potentially precipitating low cardiac output syndrome or cardiac arrest.

## 12. Conclusion

The rapid advancement in the field of TTVI is expected to drive further refinement and individualization of anesthetic management strategies. The integration of artificial intelligence and machine learning in image recognition, hemodynamic prediction, and risk stratification is becoming increasingly prevalent, offering the promise of real-time intraoperative decision support and early warning systems. Emerging technologies such as 4D intracardiac echocardiography (4D-ICE) and multimodal image fusion are set to enhance the precision of intraoperative guidance and mitigate complications. Currently, the optimal heart rate range during TTVI is largely based on the subjective experiences of operators and anesthesiologists regarding hemodynamic management; hence, further research is essential to objectively define heart rate targets. Additionally, perioperative management strategies that incorporate genetic insights into the development of right heart function, innovative pharmacological agents for right heart protection [47, 48], the utilization of mechanical circulatory support devices [49], and biomarkers indicative of right heart function [50] are anticipated to become “whole landscape” of TTVI anesthetic management.

With continuous device innovation and accumulating clinical evidence, the indications for TTVI may further expand, necessitating corresponding optimization of anesthetic management. Future efforts should include more prospective, multicenter studies to establish evidence-based anesthetic guidelines, thereby promoting the standardization and normalization of perioperative management. Through multidisciplinary collaboration and technological innovation, TTVI anesthetic management is expected to play an increasingly critical role in enhancing surgical safety and improving long-term patient outcomes.
